# Time-Resolved Fluorescence Spectroscopy of Molecularly Imprinted Nanoprobes as an Ultralow Detection Nanosensing Tool for Protein Contaminants

**DOI:** 10.3390/bios13070745

**Published:** 2023-07-19

**Authors:** Alessandra Maria Bossi, Alice Marinangeli, Alberto Quaranta, Lucio Pancheri, Devid Maniglio

**Affiliations:** 1 Department of Biotechnology, University of Verona, Strada Le Grazie 15, 37134 Verona, Italy; alice.marinangeli@univr.it; 2 Department of Industrial Engineering, University of Trento, Via Sommarive 9, Povo, 38123 Trento, Italy; alberto.quaranta@unitn.it (A.Q.); lucio.pancheri@unitn.it (L.P.); devid.maniglio@unitn.it (D.M.); 3 INFN—TIFPA, Via Sommarive 14, Povo, 38123 Trento, Italy

**Keywords:** lifetime decay, molecularly imprinted polymers, time resolved fluorescence spectroscopy, nanosensor, wine, optical sensor

## Abstract

Currently, optical sensors based on molecularly imprinted polymers (MIPs) have been attracting significant interest. MIP sensing relies on the combination of the MIP’s selective capability, which is conveyed to the polymeric material by a template-assisted synthesis, with optical techniques that offer exquisite sensitivity. In this work, we devised an MIP nanoparticle optical sensor for the ultralow detection of serum albumin through time-resolved fluorescence spectroscopy. The Fluo-nanoMIPs (∅~120 nm) were synthetized using fluorescein-O-methacrylate (0.1×, 1×, 10× mol:mol versus template) as an organic fluorescent reporter. The ability of 0.1× and 1×Fluo-nanoMIPs to bind albumin (15 fM–150 nM) was confirmed by fluorescence intensity analyses and isothermal titration calorimetry. The apparent dissociation constant (K_app_) was 30 pM. Conversely, the 10× fluorophore content did not enable monitoring binding. Then, the time-resolved fluorescence spectroscopy of the nanosensors was studied. The 1×Fluo-nanoMIPs showed a decrease in fluorescence lifetime upon binding to albumin (100 fM–150 nM), K_app_ = 28 pM, linear dynamic range 3.0–83.5 pM, limit of detection (LOD) 1.26 pM. Selectivity was confirmed testing 1×Fluo-nanoMIPs against competitor proteins. Finally, as a proof of concept, the nanosensors demonstrated detection of the albumin (1.5 nM) spiked in wine samples, suggesting a possible scaling up of the method in monitoring allergens in wines.

## 1. Introduction

Optical sensing based on synthetic artificial chemosensors has led to promising results and developments are foreseen [1,2,3,4]. Emphasis is on molecular probes that rely on a variety of chemical structures, such as host–guest macrocycles, cavitands or nucleic acids, which are exploited as chemosensors and share high affinity as a common signature. When provided with optical-responsive reporters, a quantifiable optical signal change, such as the emergence or disappearance of a spectroscopic feature, is measured upon their binding to the targeted analyte [1]. Molecular probes are exploited as standalone, or combined with nanoparticles, singly or in multiple copies, homo- or hetero-, leading to different photophysical features, and falling under the broad definition of optical nanosensors. Some polymeric biomimetics, which belong to the class of synthetic receptors, ought to be also included among the alternative and emerging category of nanosensors. In particular, molecularly imprinted polymers (MIPs) [5] are a class of synthetic receptors that provide tailor-made recognition toward a target analyte, that is conveyed by means of a template-assisted synthesis [6,7]. For the synthesis, functional monomers and crosslinkers are solvated together with the targeted analyte, this latter acting as a molecular template. Throughout the polymerization, molecular cavities stereo-chemically complementary to the template are imprinted in the nascent polymeric network. The formed MIP is able to selectively and specifically re-bind the analyte [8]. MIPs offer several advantages: they are cheap and easy to produce, possess mechanical and thermal stability and are resistant to pH extremes [9]. Recent advances in polymer synthesis permitted preparing nanometric sized MIPs (nanoMIPs) [10,11], leading to recognition materials with a higher surface-to-volume ratio. These materials were characterized by faster binding kinetics, which is a characteristic that suits sensing. MIPs have been included in a variety of optical sensor designs, ranging from optical fibers [12,13,14] to photonic structures [15] and spectroscopic readouts [16,17]. The combination of molecular imprinting, as a method for generating chemically selective binding sites, to fluorescence, as a means of signaling the presence and the concentration of a target analyte, is particularly attractive [18]. In fact, on one side, fluorescence is a widespread technique in sensing due to the high sensitivity, the low detection limits, the real-time response, and the simple format [19]. On the other side, MIP materials can easily embed fluorescent functionalities through a variety of synthetic routes [20]. Both organic fluorescent monomers, such as N-allyl-4-ethylenediamine-1,8-natphalimide, anthracene-based monomers, or 3′-Methacryloxyspirobenzo[c]-furan [1,9′]xanthen-3-one, as well as inorganics fluorescent materials, such as quantum dots (QDs), lanthanides, metal nanoclusters and upconverting nanoparticles, have been combined to the MIPs, as widely described in [17,18]. Indeed, when a fluorophore is integrated in the MIP polymer network, it effectively reports upon the binding of the analyte by changing the overall optical response. In one of the first examples, a polymerizable trans-4-[p-(N,N-dimethylamino)styryl]-N-vinyl-benzylpyridinium chloride was used as an organic fluorescent monomer in the synthesis of an MIP targeting adenosine 3′,5′ cyclic monophosphate (cAMP); the presence of cAMP was detected in aqueous solutions in the range between 10 nM and 100 μM as a quenching of the fluorescent emission [21]. Later, hybrid MIP/QDs materials were introduced [18,22,23,24]. In an example, composite nanospheres made of Mn^2+^-doped ZnS QD/MIP were prepared, showing an ability to recognize the pesticide diazinon with a linear response in the range 50–600 ng/mL [22]. Another strategy proceeded through the polymerization of MIPs thin-layer membranes, in which the fluorescence was entailed embedding L-cysteine-capped Mn^2+^-doped ZnS QDs, as reported in the case of a lysozyme-templated MIP [23]. Upon the binding of the lysozyme (100–1000 nM) to the MIP, the electron transfer between the QDs and the protein resulted in a fluorescence quenching proportional to the concentration of the analyte, addressing a limit of detection (LOD) of 10.2 nM [23].

In addition to the possibility of determining the target analyte via fluorescence amplitude changes, fluorescence decay times, or fluorescence lifetime, is an additional tool for optical sensing that is worth exploring. Lifetime measurements provide unique information about the system under consideration and have the advantage, unlike amplitude, of being considered as absolutes. In fact, for low concentration values, lifetime is a phenomenon largely independent from fluorescence intensity and/or the fluorophore concentration [25]. Moreover, fluorescence lifetime can be considered as a state function, since it does not depend on the initial perturbation conditions, such as excitation wavelength, duration of light exposure, single- or multi-photon excitation, measurement method, nor it is affected by photobleaching [26]. Despite the foreseen advantages, to date, sensing based on MIPs and fluorescence lifetime seems seriously under-explored.

Wandelt and colleagues prepared a fluorescent MIP, integrating the polymerizable trans-4-[p-(N,N-dimethylamino)-styril]-N-vinylbenzylpyridinium chloride moiety as a fluorescent reporter and using cAMP as the template [27]. The bulk MIP was ground into microparticles and used as suspension; when challenged with cAMP (10^−5^ to 10^−3^ M), the MIP microparticles showed a decrease in the fluorescence lifetime from 2.79 to 2.70 ns [27]. Using a similar recipe, fluorescent MIPs were also prepared in the form of sensing surfaces for the recognition of cAMP by the photopolymerization of an MIP thin film on a quartz support. Results showed a change in the fluorescence lifetime from 2.11 to 1.99 ns upon the binding of cAMP (10^−3^ M) [28]. Later, inorganic fluorophores based on rare earths and QDs, characterized by the advantage of extended lifetimes, were integrated into MIPs [25]. QDs were embedded into MIP silica-based microparticles of 25–30 µm in diameter and imprinted for the recognition of malachite green. Challenging these micro-MIPs with malachite green (10 µM) resulted in a decrease in the lifetime from 79 to 60 ns [29]. Quílez-Alburquerque and colleagues developed a sensor for tenuazonic acid mycotoxin (TeA) using a tailored multifunctional Ru(II) complex as a fluorescent probe [30]. An MIP nanolayer was polymerized onto 200 nm silica beads, using the trifunctional luminescent acrylate-Ru(II)-biimidazole monomer. The MIP core–shell nanoparticles had a lifetime of 72 ns, while in the presence of TeA (0.5–400 µM), a 30% decrease in the average emission lifetime was observed [30].

In the present work, we studied the effect of entailing an organic fluorescent moiety, i.e., fluorescein methacrylate, to water-soluble MIP nanoparticles (Fluo-nanoMIPs) of about 120 nm in hydrodynamic diameter that were selective for the protein human serum albumin (HSA), with the aim of devising a lifetime-based nanosensor for the assessment of protein traces. It is anticipated that Fluo-nanoMIPs nanosensors’ time-resolved fluorescent spectroscopy enabled attaining an ultralow detection of proteins, suggesting the potential for future applications in clinical, food and environmental areas.

## 2. Materials and Methods

### 2.1. Chemicals

Acrylamide (Aam), N-*tert*-butylacrylamide (tBAm), methacrylic acid (MAA), N,N′-methylene bisacrylamide (BIS), N,N,N′,N′-tetramethyl ethylenediamine (TEMED), ammonium persulfate (APS), fluorescein O-methacrylate (FluorMAA), human serum albumin (HSA, Cat. No. A9731), human transferrin (HTR, Cat. No. T3705), bovine serum albumin (BSA, Cat. No. A7906), lysozyme (Lyz, Cat. No. 10837059001), ovalbumin (Cat. No. 05440), trypsin, phosphate buffer (PB), saline phosphate buffer (PBS), tris(hydroxymethyl)-aminomethane (TRIS), 1-Ethyl-3-(3-dimethylaminopropyl) (EDC), N-hydroxysuccinimide (NHS), 2-(N-morpholino)ethane-sulfonic acid (MES), ethanol, acetonitrile and N-Cyclohexyl-2-aminoethanesulfonic acid (CHES) were from Sigma-Aldrich (Darmstadt, Germany).

### 2.2. Synthesis of Fluo-nanoMIPs

The synthesis of the fluorescent nanoMIPs (Fluo-nanoMIPs) was carried out using a total monomer concentration of 0.2% (*w*/*v*). The monomers Aam, MAA and tBAm were used in a ratio 8, 8 and 4% moles, respectively, and admixed to 80% (moles) of BIS in 20 mM PB at pH 7.4, as described in [16]. Fluorescence was entailed adding fluorescein methacrylate in a quantity of 1.3, or 13, or 130 nmol with respect to the total monomers. The template, HSA, was 15 nmol. Vials were closed with rubber caps and bubbled with N_2_ for 10 min. The catalysts, APS (0.04% *w*/*v*) and TEMED (0.03% *w*/*v*), were added and the polymerization was carried out overnight at room temperature under mild stirring. At the completion of the polymerization, the removal of the template was carried out by enzymatic digestion with trypsin (100 µg) for 2 h at 37 °C, which was followed by dialysis (M.W.C.O. 14.000 Da, Sigma-Aldrich, Darmstadt, Germany) with 3 × 3 L of MilliQ water. Next, the Fluo-nanoMIPs were freeze-dried and stored. The yield of polymerization was 85%, as estimated from the weight of the lyophilized nanoparticles with respect to the total weight of the monomers used in the synthesis.

### 2.3. Calibration Curve for FluorMAA

The fluorescence intensity of increasing concentrations of FluorMAA (81.25, 162.5, 325, 650 and 1300 nM) was measured using a spectrofluorometer (FP-8200, Jasco Ltd., Heckmondwike, UK) with an excitation wavelength at 488 nm and reading the emission intensity at 514 nm, corresponding to the emission peak. The calibration curve for FluorMAA and the associated equation are reported in Supplementary Section S1. The fluorescence intensities at the emission wavelength λ_em_max_ = 514 nm of the Fluo-nanoMIPs samples (1 mg/mL) were measured, and the calibration was used to estimate the amount of fluorophore incorporated within the polymeric network during the synthesis. Measurements were in triplicate.

### 2.4. Dynamic Light Scattering (DLS)

Size distribution and the polydispersity index (PDI) were determined using a Zetasizer Nano ZEN3600 (Malvern Instruments Ltd., Malvern, UK) equipped with a 633 nm He-Ne laser at a detection angle of 173°. Fluor-nanoMIPs were suspended in water to the final concentration of 1 mg/mL. The material refractive index (RI) was 1.490 and the absorption value was 0.01; the dispersant RI was 1.332 for water, while the viscosity was 0.89 cP as reported by the Zetasizer V.6.32 software (Malvern instruments Ltd., Malvern, UK). The temperature was set at 298° K. Measurements were in triplicate.

### 2.5. Scanning Electron Microscopy (SEM)

Images were collected with a secondary electron detector at 15 keV beam energies on a Supra 40 Field Emission SEM (Zeiss, Oberkochen, Germany). Prior to SEM analysis, samples were suspended in ultrapure water at 150 µg/mL final concentration and briefly ultrasonicated. Then, 5 μL of the suspension were deposited on a silicon wafer substrate mounted with carbon double tape on an aluminum stub, after which it was dried at 60 °C for 24 h or 30 °C for 72 h. A platinum/palladium ultrathin coating (2 nm) was deposited by means of plasma sputtering to ensure electrical conductivity.

### 2.6. Fluorescence Intensity of Fluo-nanoMIP

Fluo-nanoMIPs, respectively, synthetized with 1.3, 13 and 130 nmol of FluorMAA, were dissolved at 0.2 mg/mL and incubated with increasing concentrations (from 15 fM to 150 nM) of human serum albumin, or with the same concentrations of human serum transferrin, i.e., a non-template protein chosen for selectivity tests. The equilibrium time, previously tested over 60 min, was reached in 20 min. After 30 min of incubation, samples were plated in triplicate on a 96-well plate hydroGrade (BRANDplates, Germany) with a volume of 60 μL per well. Steady-state measurements of Fluo-nanoMIPs were carried out using the microplate reader Infinite 200 PRO (Tecan Group Ltd., Männedorf, Switzerland) at the emission wavelength of 522 nm. Emission intensities were measured as relative fluorescent units (rfu). Isotherm was fitted with OriginPro 9.0 using the Langmuir model equation: y = START + (END − START) × x/(k + x), where START and END were the initial and final y values; x was the concentration of HSA; and k was the half-saturation or apparent dissociation constant (EC_50_ or apparent dissociation constant K_app_).

### 2.7. Fluorescence Lifetime of Fluo-nanoMIP

The fluorescence lifetime of a population, measured in the time-domain, also called fluorescence intensity decay, follows the equation:

(1) It=I0e−tτ

where $I\left(t\right)$ is the intensity at time t; $I_{0}$ is the intensity at *t* = 0; *t* is the time after the absorption; and τ is the fluorescence lifetime. Time-resolved fluorescence intensities were collected using a single photon counting spectrometer Nanolog/Fluorolog-3-2iHR320 (Horiba-Jobin Yvon, Kyoto, Japan) equipped with a NanoLED source with a wavelength of 453 nm. The emission was monitored at the angle of 90° with respect to the excitation. Data were collected in 1023 channels to 10,000 counts in the peak, while the calibration time was 109.73 ps per channel. The voltage at the photomultiplier (PTM) was set to 950 V. Measurements were performed in a 1 mL quartz cuvette, using a fixed concentration of 0.2 mg/mL of nanoMIPs in PBS (10 mM pH 7.4), adding increasing concentrations of HSA (100 fM–150 nM). To allow binding kinetics’ stabilization, a 20 min incubation was awaited before performing each measurement.

A 0.2 mg/mL Fluo-nanoMIP suspension was excited at λ_exc_ = 453 nm to obtain the instrumental response (prompt) for the deconvolution. The sample decays were recorded at λ_em_ = 522 nm.

Data were elaborated with the Decay Analysis Software V. 6.8 (Horiba Scientific, Yvon, Kyoto, Japan), choosing a biexponential fitting equation model:

(2) $$I\left(t\right)=A+B_{1}e^{-t/\tau 1}+B_{2}e^{-t/\tau 2}$$

The biexponential fitting was used to take into account the heterogeneity of the system due to the random incorporation of the fluorophore in the Fluo-nanoMIPs polymeric network during the synthesis. The two components, $\tau_{1}$ and $\tau_{2}$ , are used to describe the presence of two different populations of fluorophores: one integrated inside the stamped molecular cavities and the other outside the binding cavities, randomly distributed in the nanoMIP, according to [30,31].

### 2.8. Sensor Parameters

The lifetime values ( $\tau_{2}$ ), plotted as a function of HSA concentration, described the binding isotherm for the sensing system. Data were fitted with the Hill equation model (OriginPro 9.0):

(3) $$\tau_{2}=\tau_{2\_max}\frac{x^{n}}{\left(K+x^{n}\right)}$$

where $\tau_{2}$ is the lifetime at concentration x of the ligand (i.e., HSA); $\tau_{2}$ __max_ is the τ value at binding saturation; n is the Hill parameter, which correlates with the number of binding sites, and *K* is the apparent dissociation constant derived from the law of mass action, which is represented by the following equation:

(4) $$K=\frac{\left[A\right]\cdot \left[B\right]}{\left[AB\right]}$$

where [ A ] is the molar concentration of the receptor which in our case consists of the 1×Fluo-nanoMIPs; [ B ] is the molar concentration of the analyte and [ AB ] is the concentration of the complex between the 1×Fluo-nanoMIPs and the analyte.

### 2.9. Selectivity of 1×Fluo-nanoMIPs

Different competitor proteins were chosen for the selectivity of fluorescent nanoMIPs. A solution of 1×Fluo-nanoMIPs at 0.2 mg/mL was incubated for 20 min in the presence of 18 pM of BSA, or 20 pM of HTR, or 11 pM of ovalbumin, or 17 pM of lysozyme. Fluorescence lifetime was measured as explained above. Measurements were performed in triplicate.

### 2.10. Fluorescence Lifetime of 1×Fluo-nanoMIPs in Wine

Measurements were performed in a 1 mL quartz cuvette, using chardonnay white wine, spiked with 1.5 nM of serum albumin, diluted 1:5 in PBS 10 mM pH 7.4 into which 1×Fluo-nanoMIPs were dispersed at a concentration of 0.2 mg/mL. In this case, the prompt was prepared with the same wine dilution and nanoparticles but without the spike and recorded according to Section 2.8. Selectivity was tested using the same concentration of human transferrin. Measurements in triplicate were performed as described above.

### 2.11. Isothermal Titration Calorimetry (ITC)

Isothermal titration calorimetry (ITC) was performed on a MicroCal PEAQ-ITC (Malvern Panalytical Ltd., Worcestershire, UK) instrument. All solutions were filtered and degassed prior to use. The 1×Fluo-nanoMIPs, human serum albumin and human serum transferrin (HTR) were solubilized in 10 mM PBS pH 7.4. Then, 1 μM of 1×Fluo-nanoMIPs (200 µL) was titrated with 30 nM to 10 μM of serum albumin at 25 °C, and the heats of the interactions were recorded. Dilution heats were estimated from the titration of the proteins in buffer. Raw heats were subtracted from the dilution heat and integrated. Integrated heats were plotted as a function of the molar ratio between the titrand and the titrant and fitted with one set of sites modeled with the MicroCal PEAQ-ITC Analysis Software 1.22.1293 to estimate values for the binding constant (K_D_) and the enthalpy variation associated with binding (ΔH).

### 2.12. Atomic Force Microscopy (AFM)

For the AFM, nanoMIPs were covalently coupled to support surfaces with the protocol reported in [14]. The surface topography of the of Fluo-nanoMIPs was studied using a NT-MDT Solver Pro system equipped with a Nova scanner. Samples were imaged in semi-contact mode using super sharp diamond-like carbon tips (NSG01_DLC, NT-MDT, 1 nm nominal tip radius, 150 kHz, force constant 5.5 N/m), collecting 1 × 1 µm, 512 points resolution topography images. AFM data were analyzed with the support of Gwyddion analysis software [32].

## 3. Results and Discussion

### 3.1. Effects on the Lifetime Due to Interaction between the Fluorophore and Albumin

**Figure 1:**
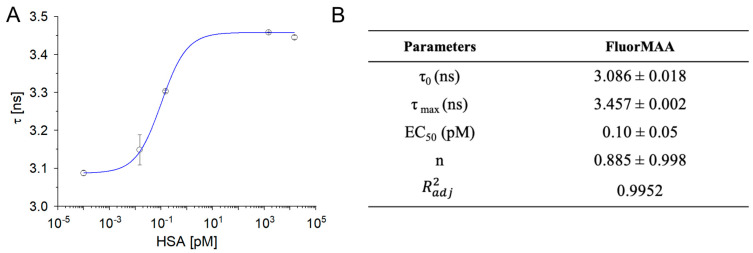
(**A**) Fluorescence lifetime of FluorMAA as a function of albumin concentration (n = 3) fitted with Hill model equation. (**B**) Fitting parameters of the fluorescent decays of FluorMAA in the presence of albumin (monoexponential model, SI Section S2).

Initially, the effects on the fluorophore lifetime due to the interaction between the chosen organic fluorescent monomer (FluorMAA) and the target analyte was studied by time-resolved fluorescent spectroscopy (SI Section S2). FluorMAA (155 pmol) was incubated with increasing concentrations of albumin (15 fM–15 nM), and the lifetimes ( τ ) were measured (Figure 1).

The lifetime increased with the albumin concentration following a sigmoidal profile, with values shifting from 3.086 ± 0.018 to 3.457 ± 0.002 ns and plateauing for protein concentrations around 10 pM (Figure 1; fitting parameters in the inset table). Such a behavior was indicative of an interaction taking place between the fluorophore and the protein. In line with previous observations, increments of τ values of the protein’s endogenous reporters (tryptophans) were observed in the case of non-specific interactions with the environment, such as bovine serum albumin adsorbing to ZnO core–shell nanoparticles [33].

### 3.2. Synthesis and Characterization of Fluorescent NanoMIPs

**Figure 2:**
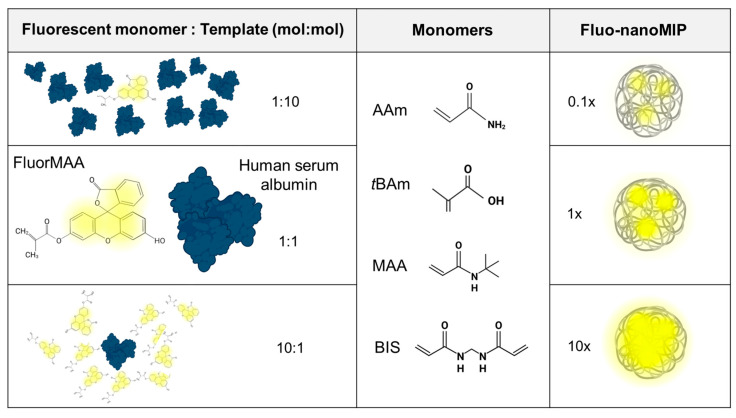
Scheme of the polymerization conditions used to prepare the library of Fluo-nanoMIPs.

A library of fluorescent nanoMIPs, herein called Fluo-nanoMIPs, was synthesized using a total monomer concentration of 0.2% *w*/*v*. In particular, acrylamide (Aam), *tert*-butylacrylamide (tBAm), and methacrylic acid (MAA) were admixed to N,N’-methylene bis-acrylamide (BIS), used as a reticulating agent, as reported in [14]. Human serum albumin (15 nmol) was chosen as a model template and added to the pre-polymerization mixture (V_final_ = 10 mL) [34]. According to the scheme reported in Figure 2, fluorescence was entailed by using the fluorescein–methacrylate (FluorMAA) monomer, which was added to the syntheses at 1.3, or 13, or 130 nmol to the final synthetic volume, in order to study the effect of the molar ratio between the fluorescent-reporter (0.1×, 1×, 10×) and the template on the fluorescent readouts (Table 1). At the completion of the syntheses, the degree of incorporation of the fluorophore into the MIP nanoparticles was estimated by means of the calibration curve: y (λ_em_@522 nm) = 0.82 × ([FluorMAA], nM) − 0.12 (details in SI Section S1 Calibration Curve). Results indicated the FluorMAA incorporation for the 0.1×, 1× and 10×FluorMAA was, respectively, 28, 23 and 52% (Table 1).

**Figure 3:**
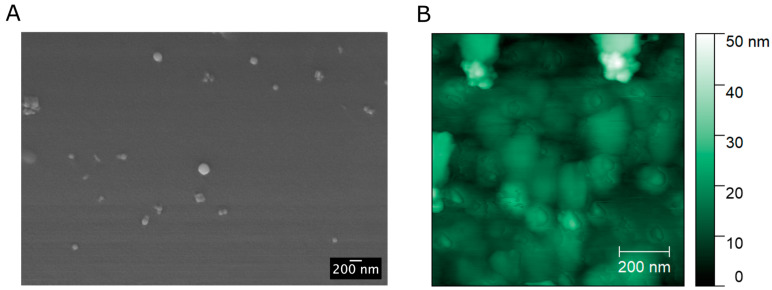
(**A**) Exemplificative SEM image of Fluo-nanoMIPs; (**B**) AFM image of Fluo-nanoMIP covalently coupled to silica supports.

The Fluo-nanoMIPs dimensions were characterized by means of dynamic light scattering (DLS), and the estimated sizes are reported in Table 1. The hydrodynamic sizes of the nanoparticles were about a hundred nanometers, while the polydispersity index (PDI) indicated a homogeneous distribution. As a confirmation, Figure 3 reports the scanning electron microscopy (SEM) and atomic force microscopy (AFM) images of the Fluo-nanoMIPs, showing spherical nanoparticles with dimensions in agreement with DLS data.

### 3.3. Functional Characterization of the Fluo-nanoMIPs

#### Study of the Fluorescence Intensity of the Library of Fluo-nanoMIPs upon Binding

**Figure 4:**
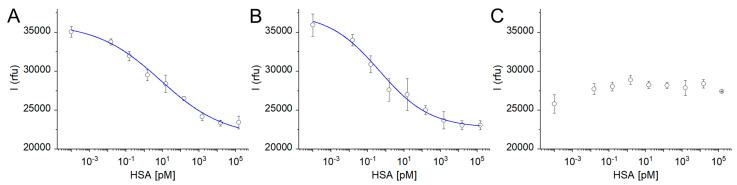
Emission intensity at 522 nm of: (**A**) 0.1×Fluo-nanoMIP; (**B**) 1×Fluo-nanoMIP; (**C**) 10×Fluo-nanoMIP challenged with albumin.

The functional characterization of the library of Fluo-nanoMIPs was assessed by means of fluorescent intensity binding studies. Each batch of Fluo-nanoMIPs, i.e., 0.1×, 1× and 10×, was incubated for 20 min with increasing concentrations of HSA (15 fM–150 nM), and the fluorescence emission at 522 nm was monitored. Quenching of the emission intensities was observed both for the 0.1× and the 1×Fluo-nanoMIPs challenged with increasing concentrations of albumin (Figure 4A,B). Binding data were fit with a Langmuir equation model, and the resulting parameters are reported in Table 2. Worth of note is the value of the half saturation, EC_50_, which corresponds to the apparent dissociation constant (K_app_), which was estimated in the pM range, indicating a remarkably high affinity of the imprinted nanomaterial for its targeted protein. Both the tested quantities of fluorescent reporter, i.e., 0.1× or 1× moles with respect to the moles of the template, yielded to Fluo-nanoMIPs sensitive to binding events in the range between the fM and the pM, indicating these nanosensors’ compositions were apt to detect traces of the protein template. In contrast, in the case of the 10×Fluo-nanoMIPs, no variation in the emission intensity was observed upon albumin addition (Figure 4C). This supported the hypothesis that a synthetic condition, in which the fluorophore reporter is added in molar excess with respect to the template protein, does result in several fluorescent labels per nanoparticle being randomly distributed in the polymeric network. Such a condition likely includes the placement of fluorophores outside of the formed binding sites, together with self-quenching effects, making the overall fluorescence response non-sensitive to the binding events.

Therefore, 0.1× and 1×Fluo-nanoMIPs demonstrated the crucial effect of a careful adjustment of the quantity of fluorescent reporter (in moles) with respect to the moles of the template (i.e., 1:10 and 1:1). Both compositions relied on a restricted number of fluorescent tags per molecule of albumin in the pre-polymerization mix, which was a condition hypothesized to favor the fluorophore to albumin pairing during the pre-synthetic stage, and ultimately leading to a superior control of the placement of the few fluorescent reporters in, or close by, the binding cavity, in the formed nanomaterials. Yet, the best fluorescent performance at binding was observed for the composition based on a one-to-one fluorophore to albumin molar ratio, as shown by the steeper slope of the fitting curve reported in Figure 4B when compared to 4A. It appears that the strategy to modulate the number of fluorescent tags on the nanoMIPs as a function of the quantity of template used in the synthesis is very straightforward and easy to perform yet lacking the fidelity that can be achieved by the post-synthetic chemical tagging of MIPs with fluorescent probes, as reported for the post-imprinting approach proposed by [35,36].

**Figure 5:**
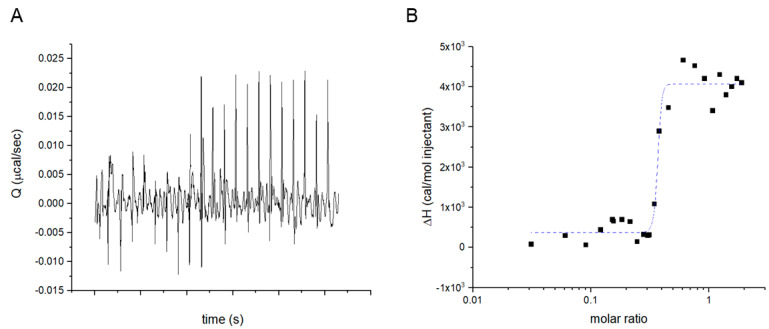
Isothermal titration nanocalorimetry data of 1×Fluo-nanoMIP titrated with human serum albumin: (**A**) raw heats over time; (**B**) solid squares, integrated heats fitted with a one-site equation model for the titration with serum albumin with 1×Fluo-nanoMIPs.

As an independent proof, the specific binding between albumin and the synthesized 1×Fluo-nanoMIPs was assessed by isothermal titration nanocalorimetry (ITC) [37]. The interaction between the nanomaterial and its targeted analyte is reported in Figure 5. As shown in Figure 5B, the integrated heats described a sigmoidal profile, which is typically associated with interacting molecules. The steep transition is typical of a binding event characterized by extremely high affinity; in fact, the dissociation constant was in the pM range. The ITC confirmed the effective stamping of binding sites on the 1×Fluo-nanoMIPs [37,38]. In contrast, when 1×Fluo-nanoMIPs were titrated with a non-template protein, no interaction was observed (SI Section S4 Isothermal Titration Calorimetry).

### 3.4. Fluorescence Lifetime of the Fluo-nanoMIPs

**Figure 6:**
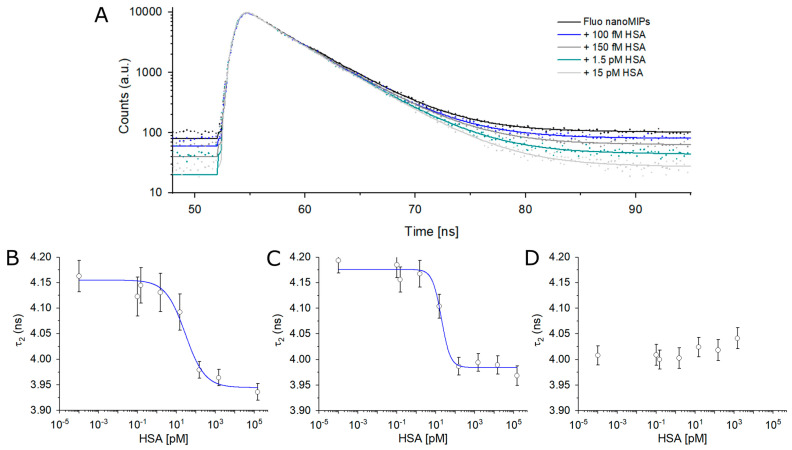
(**A**) Lifetime fluorescence spectra of Fluo-nanoMIPs in the presence of increasing concentrations of albumin. Fluorescence lifetime ( $\tau_{2}$ ) of (**B**) 0.1×Fluo-nanoMIP, (**C**) 1×Fluo-nanoMIP and (**D**) 10×Fluo-nanoMIP as a function of albumin concentration. Binding curves were fitted with Hill model equation.

The feasibility of devising soluble nanosensors, based on Fluo-nanoMIPs, to monitor the presence of serum albumin, as the target analyte, by fluorescence lifetime decay was next investigated. The intensity decay curve of Fluo-nanoMIPs (0.2 mg/mL) was recorded at λ_em_ = 522 nm, allowing the samples to equilibrate for 20 min. Data were fitted with a biexponential fitting ( $\tau_{1}$ and $\tau_{2}$ ). Indeed, this was an approximate, though acceptable [33], model to represent and discriminate between the decay’s contributions given by the fluorescent reporters randomly placed on the polymer backbone and the ones related to effectively placed fluorescent reporters, i.e., located within, or nearby, the imprinted binding site in the Fluo-nanoMIPs. In detail, a fixed $\tau_{1}$ was used as a global descriptor of all the non-specific decays occurring outside the molecularly imprinted cavities, whereas the binding-related interactions were observed as $\tau_{2}$ . It appeared that both solvated 0.1× and 1×Fluo-nanoMIPs in the absence of the analyte displayed very similar $\tau_{2}$ values, i.e., 4.154 ± 0.015 and 4.183 ± 0.009 ns, respectively, which is in agreement with the theory of lifetime decay that postulates the independence of the τ value from the concentration of fluorophore at low concentrations. Additionally, we observed a significative difference in the τ values of free FluorMAA (Figure 1) with respect to τ of the fluorophore incorporated in the nanoMIP (Table 3) due to the different environment surrounding the molecule and accounting for its shielded integration within the nanoMIP. In contrast, the 10×Fluo-nanoMIPs, characterized by evenly distributed reporters and/or self-quenching effects, showed significantly lower values of τ . It can be expected that the more the fluorophores on the nanoMIPs are freely exposed to the solvent, the more the τ value should approach that of the free Fluor-MAA. Overall, the differences in fluorophore decays observed by comparing the 0.1× and 1×, versus 10×Fluo-nanoMIPs, were evidence of the strong correlation between the decay’s properties and the fluorescent reporter’s placement in the nanoMIP’s backbone [26]. Next, Fluo-nanoMIPs (0.1×, 1×, and 10×) were solvated in PBS at the concentration of 0.2 mg/mL and challenged with increasing concentrations of albumin, from 100 fM to 150 nM. The results are reported in Figure 6. Characteristics saturation binding isotherms were observed for 0.1× and 1×Fluo-nanoMIPs (Figure 6B,C), whereas 10×Fluo-nanoMIPs did not report any response to binding events (Figure 6D). Table 3 reports the data of the binding fitted with the Hill equation model.

Concerning 0.1× and 1×Fluo-nanoMIPs, the nanosensor’s half saturation was in the pM range, which was a value that remained consistent with the observations reported for the fluorescent intensity experiments (Table 2). Fitting parameters showed how an n value, which in this case correlates with the number of optically active binding sites, increased from 0.86 to 1.89 when nanoMIPs were prepared with 10 times more of the fluorescent reporter. This suggested that the 1×Fluo-nanoMIPs should be preferred for sensing.

The sensor’s operational parameters in PBS, associated to the 1×Fluo-nanoMIP nanosensors, were then extrapolated and are reported in Table 4, indicating that the nanomaterial herein prepared can be exploited to determine serum albumin in the picomolar range.

### 3.5. Selectivity of the 1×Fluo-nanoMIP Nanosensor

**Figure 7:**
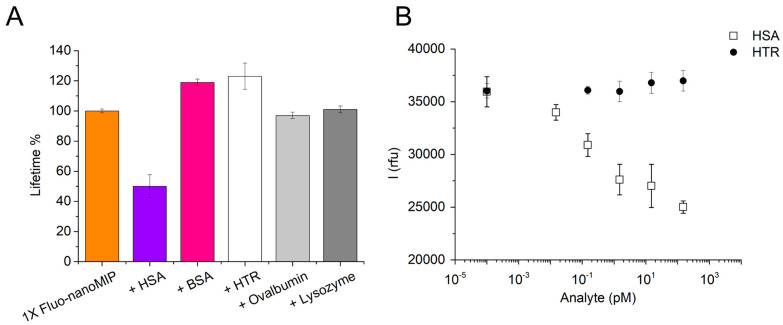
(**A**) Selectivity of 1×Fluo-nanoMIPs studied by time-resolved fluorescence spectroscopy. Orange bar reports τ_2_ of a sample with just 1×Fluo-nanoMIPs, in purple τ_2_ for 1×Fluo-nanoMIPs incubated with HSA (18 pM) and compared with pink for τ_2_ of the same Fluo-nanoMIPs incubated with bovine serum albumin (18 pM); white for τ_2_ of HTR (20 pM); light gray for τ_2_ of ovalbumin (11 pM); or dark gray for τ_2_ of lysozyme (17 pM). (**B**) For a better comparison, the selectivity of 1×Fluo-nanoMIPs was studied in terms of emission intensity at λ_max_= 522 nm. Open squares represent 1×Fluo-nanoMIP incubated with the targeted HSA; solid circles represent 1×Fluo-nanoMIP incubated with the competitor HTR.

Finally, a selectivity test was carried out to confirm that the variations in lifetime decays reported in Figure 6 were due to specific binding events. The selectivity test was per-formed by choosing different proteins as interferents. Bovine serum albumin (BSA), which is characterized by molecular weight (MW 66,000 g/mol) and isoelectric point (pI 6.8) similar to HSA, was selected to epitomize the ability of Fluo-nanoMIPs to bind with other mammalian albumins. Hen egg ovalbumin (MW 42,700 g/mol, pI 5.1) was chosen for testing the selectivity toward an albumin from a different species (*Gallus gallus*), whereas the hen egg lysozyme that is characterized by a higher pI and a significantly smaller size (MW 14,400 g/mol, pI 9.36) was chosen for devising the effect of charge on the Fluo-nanoMIP binding. Finally, another highly abundant serum protein, namely human serum transferrin (HTR, MW 77,000 g/mol, pI 6.8), was also tested. As shown from the histogram chart reported in Figure 7A, the incubation of the nanosensors with HSA (18 pM) produced a 40% drop in the τ_2_ value. In contrast, the nanosensors in the presence of any of the non-template proteins did not produce significant changes in the lifetime decays.

In the case of BSA and of HTR, a slight increment in lifetime was observed, which was possibly ascribed to non-specific interactions between the interferent protein and the biomimetic nanomaterial [33]. As an independent proof, the specificity of the 1×Fluo-nanoMIP nanosensors was also tested by comparative binding, in fluorescence emission, indicating the effect of increasing concentrations of HSA (Figure 7B, open squares) or of the HTR (Figure 7B, solid circles). Results confirmed quenching of the emission solely associated to the binding of, or occurring nearby, the optical-responsive reporters of the nanosensor, whereas no emission changes were observed when the HTR interferent was tested. This confirmed the selectivity of the 1×Fluo-nanoMIPs and supported their use as soluble nanosensors for albumin detection.

### 3.6. Fluo-nanoMIP Nanosensors for the Determination of Albumin Allergen in Wine Samples

**Figure 8:**
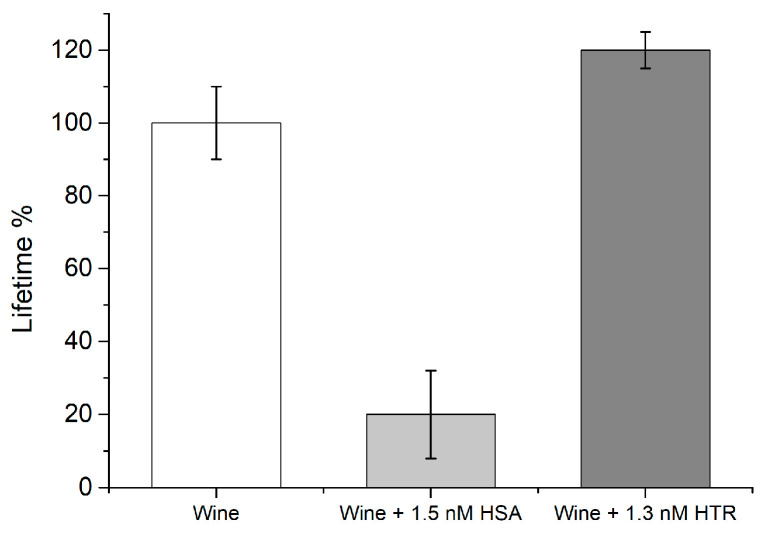
Real sample testing by means of 1×Fluo-nanoMIP nanosensors. White bars: wine sample; Gray bar: wine sample spiked with a known concentration of albumin; Dark-gray bar: wine sample spiked with a known concentration of HTR as an example of unrelated protein.

The sensitivity of the herein synthesized 1×Fluo-nanoMIP nanosensors supported their use in real scenarios in order to determine the albumin contamination at ultralow concentrations, such as in the case of protein allergen traces in wines [39]. In a preliminary experiment, we tested the use of the nanosensors in real wine samples (n = 2) that were spiked with albumin (1.5 nM), whereas control samples were both not spiked and spiked with the non-related protein HTR. All samples (final volume of 1 mL) were supplied with a fixed quantity of nanosensors (0.2 mg/mL), incubated for 20 min and measured. Figure 8 reports the measured lifetime decays. It was observed that the percentage of the $\tau_{2}$ value dropped exclusively when in the presence of albumin, confirming the selectivity of the nanosensors.

## 4. Conclusions

The fluorescence lifetime is an intrinsic property of fluorescent probes that is highly sensitive to the microenvironment [40], while it is largely independent from both the fluorescence intensity and the fluorophore concentration; therefore, it can be effectively used as a reporter of molecular interactions. In the present work, we exploited an organic fluorophore monomer (Fluor-MAA) to synthesize analyte-selective fluorescent polymeric nanosensors by means of the MIP technology. A known nanoMIP composition, suitable to recognize HSA [14], was entailed of the fluorescent reporter Fluor-MAA, and the binding was studied through fluorescence decay, which was a yet not-investigated response for the system. Valuable information about the binding event was reported as a decrease in the lifetime decays. It was observed that sub- to stoichiometric quantities of Fluor-MAA with respect to the albumin template, i.e., 0.1× and 1×Fluo-nanoMIPs, appear to statistically guide the placement of the fluorescence probe within the imprinted binding cavity, allowing to determine the presence of the target protein at picomolar levels, which is a limit significantly improved over the current literature [30]. These achievements contribute to the advancement of nanosensing by means of the MIP technology and to the in-solution real-time determination of protein markers [4].

Currently, the interest in the use of nanoMIPs is mainly in clinical diagnostics, but expected areas of application concern the detection of traces of proteins that are causative agents of allergies in foods. Along the way to develop nanosensors for allergies, we started by studying the Fluo-nanoMIP’s ability to monitor ultralow quantities of the model protein HSA in the wine matrix. The results were positive, opening to the further synthesis and to further lifetime interrogations of Fluo-nanoMIPs tailored to the specific allergenic proteins found in wines (i.e., lysozyme, ovalbumin) [41]. From the present results, it appeared that Fluo-nanoMIPs can constitute a general answer to the growing need for ultralow sensitive analytical devices for the determination of protein contaminations in beverages [42]. Additionally, we foresee the possibility to devise multiple Fluo-nanoMIPs, each templated toward the recognition of a specific allergenic target, expanding the current MIP sensing portfolio. In view of a multi-targeting nanosensing and to their translation to the market, it would be beneficial to explore the solid-phase synthesis approach, which is compatible to mass production of the Fluo-nanoMIPs [11,43].

## Figures and Tables

**Table 1:** Fluorescence and physical characteristics of the nanoMIPs.

Sample Name	FluorMAA Added to Polymerization (pmol/mg)	FluorMAA Incorporated (pmol/mg)	Z_average_ (nm)	PDI
0.1×Fluo-nanoMIP	65	20 ± 5	115.6 ± 0.8	0.20
1×Fluo-nanoMIP	650	155 ± 20	123.9 ± 0.1	0.22
10×Fluo-nanoMIP	6500	3380 ± 300	176.6 ± 0.4	0.34

**Table 2:** Fitting parameters of emission intensity measurements of Fluo-nanoMIPs 0.1×, 1× and 10× incubated with increased concentrations of albumin.

Parameters	0.1×Fluo-nanoMIP	1×Fluo-nanoMIP	10×Fluo-nanoMIP
I_0_ (rfu)	33,578 ± 535	34,399 ± 476	25,805 ± 1175
I_min_ (rfu)	23,452 ± 547	23,103 ± 282	28,890 ± 573
EC_50_ (pM)	65 ± 20	30 ± 9.5	n.a. *
$R_{adj}^{2}$	0.9690	0.9883	n.a.

* Fitting did not converge.

**Table 3:** Fitting parameters of fluorescence lifetime measurements of Fluo-nanoMIPs 0.1×, 1× and 10× incubated with increased concentrations of albumin.

Parameters	0.1×Fluo-nanoMIP	1×Fluo-nanoMIP	10×Fluo-nanoMIP
τ_2_0_ (ns)	4.154 ± 0.015	4.183 ± 0.009	4.008 ± 0.018
τ_2_max_ (ns)	3.944 ± 0.009	3.983 ± 0.006	4.000 ± 0.018
EC_50_ (pM)	28 ± 13	18 ± 4.2	n.a. *
n	0.86	1.89	n.a.
$R_{adj}^{2}$	0.9678	0.9803	n.a.

* Fitting did not converge.

**Table 4:** Parameters for the 1×Fluo-nanoMIP lifetime nanosensor.

τ_2_0_ (ns)	4.183 ± 0.009	
τ_2_max_ (ns)	3.983 ± 0.006	
K_app_ (pM)	18 ± 4.2	
K_aff_ (M^−1^)	1.4 × 10^10^	Kaff = 1/*K* (M^−1^)
LOD (pM)	1.26	3 × std.dev_blank_/Sensitivity_low conc_
Sensitivity at low concentration	7.14 × 10^9^	$|{\Delta \tau }_{2\_\max}-{\Delta \tau }_{2\_0}|/K$
$\chi_{\mathrm{red}}^{2}$	0.941	
Linear dynamic range (pM)	3.0–83.5	10–90%

## Data Availability

The data are available on reasonable request from the corresponding author.

## Supplementary Materials

The following supporting information can be downloaded at: https://www.mdpi.com/article/10.3390/bios13070745/s1, Figure S1: Calibration curve of FluorMAA; Table S1: Equation parameters of the FluorMAA calibration curve; Figure S2: Examples of DLS measurements of (A) 0.1× and (B) 1×Fluo-nanoMIPs; Figure S3: Isothermal titration nanocalorimetry data of 1×Fluo-nanoMIP titrated with the non-template protein human serum transferrin and expressed as raw heats over time.
