# Correction: Bossi et al. Time-Resolved Fluorescence Spectroscopy of Molecularly Imprinted Nanoprobes as an Ultralow Detection Nanosensing Tool for Protein Contaminants. *Biosensors* 2023, *13*, 745

**DOI:** 10.3390/bios16030141

**Published:** 2026-02-28

**Authors:** Alessandra Maria Bossi, Alice Marinangeli, Alberto Quaranta, Lucio Pancheri, Devid Maniglio

**Affiliations:** 1 Department of Biotechnology, University of Verona, Strada Le Grazie 15, 37134 Verona, Italy; alice.marinangeli@univr.it; 2 Department of Industrial Engineering, University of Trento, Via Sommarive 9, Povo, 38123 Trento, Italy; alberto.quaranta@unitn.it (A.Q.); lucio.pancheri@unitn.it (L.P.); devid.maniglio@unitn.it (D.M.); 3 INFN—TIFPA, Via Sommarive 14, Povo, 38123 Trento, Italy

## Error in Figure

In the original publication [1], there was a mistake in Figure 8. Specifically, the label reported in the grey bar on the *x*-axis as “Wine + 1.5 nM BSA” was incorrect and should be “Wine + 1.5 nM HSA”. The corrected Figure 8 appears below. The authors state that the scientific conclusions are unaffected. Moreover, in the originally published version, the vertical axis was labelled as τ_2_ (ns), which could be misleading. Although τ_2_ refers to a fluorescence lifetime component, the plotted values represent the relative variation expressed as a percentage contribution of the lifetime, rather than an absolute lifetime value in nanoseconds. For this reason, the vertical axis label has been revised in the corrected version as “Lifetime %” to indicate the percentage of lifetime variation, in order to accurately reflect the nature of the reported data.

These corrections were approved by the Academic Editor. The original publication has also been updated.

## Figures and Tables

**Figure 8:**
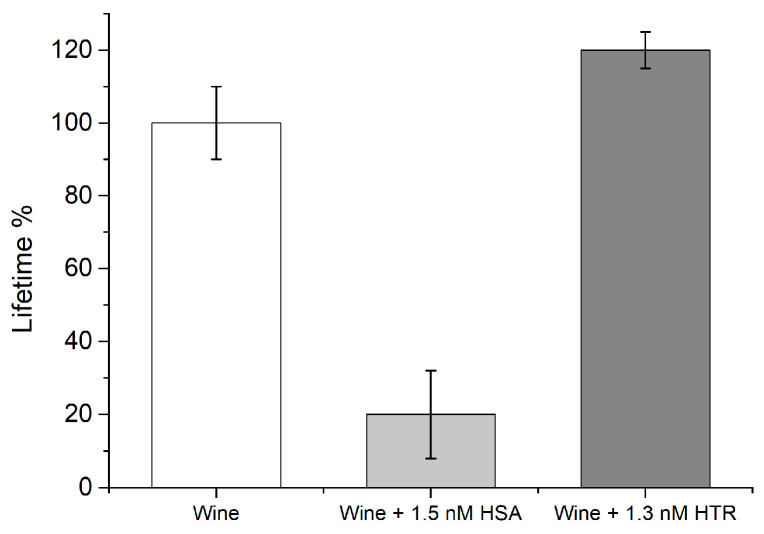
Real sample testing by means of 1×Fluo-nanoMIP nanosensors. White bars: wine sample; Gray bar: wine sample spiked with a known concentration of albumin; Dark-gray bar: wine sample spiked with a known concentration of HTR as an example of unrelated protein.

## References

[1] Bossi A.M., Marinangeli A., Quaranta A., Pancheri L., Maniglio D. (2023). Time-Resolved Fluorescence Spectroscopy of Molecularly Imprinted Nanoprobes as an Ultralow Detection Nanosensing Tool for Protein Contaminants. Biosensors.

