# Supplementary material for: Time-Resolved Fluorescence Spectroscopy of Molecularly Imprinted Nanoprobes as an Ultralow Detection Nanosensing Tool for Protein Contaminants
Source: Biosensors (Basel). 2023 Jul 19;13(7):745. doi: 10.3390/bios13070745 (PMC10377162; doi:10.3390/bios13070745)

Supplementary information

Time resolved fluorescence spectroscopy of molecularly imprinted nanoprobes as an ultralow detection nanosensing tool for protein contaminants.

1. **Calibration curve FluorMAA**

Figure S1. Calibration curve of FluorMAA

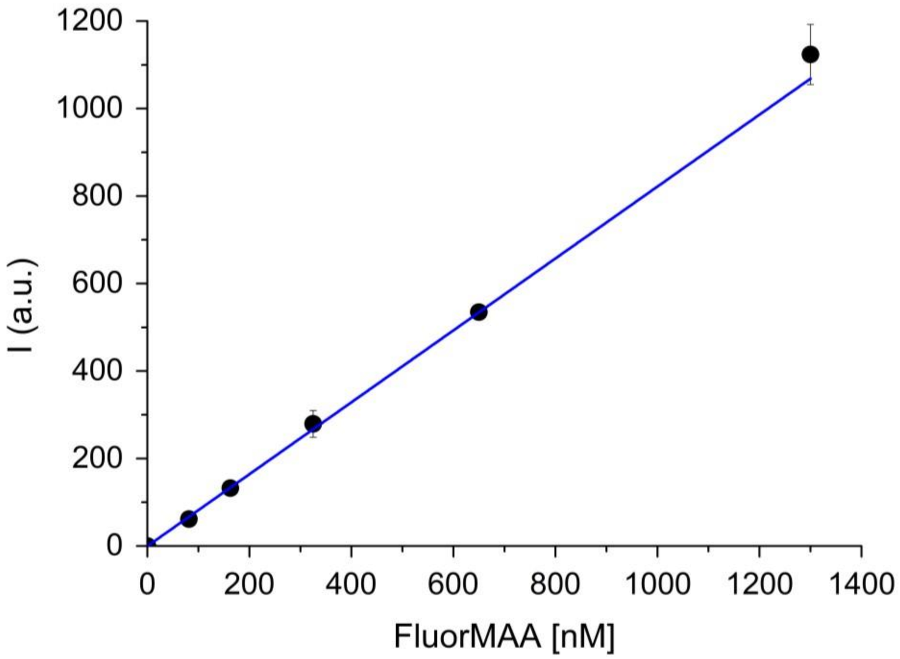

Table S1. Equation parameters of the FluorMAA calibration curve.

Equation	y = a + bx
Intercept (a)	-0.1273
Slope (b)	0.8219
R square	0.9999

2. Fluorescence lifetime of FluorMAA

The time-resolved fluorescence decays were collected as described in Section 2.7. Measurements were performed in a 1 mL quartz cuvette, using a fixed concentration of 155 pmol of FluorMAA in PBS (10 mM pH 7.4), adding increasing concentrations of HSA (15 fM – 15 nM). To allow binding kinetics' stabilization, a 20 min incubation was awaited before performing each measurement.

Because the fluorophore was freely solvated in solution, data were elaborated with the Decay Analysis Software v. 6.8 (Horiba Scientific), choosing the monoexponential fitting equation model:

$$I(t) = A + B_1 e^{-t/\tau 1}$$

3. Dynamic light scattering (DLS) of Fluo-nanoMIPs

Figure S2. Examples of DLS measurements of (A) 0.1× and (B) 1×Fluo-nanoMIPs.

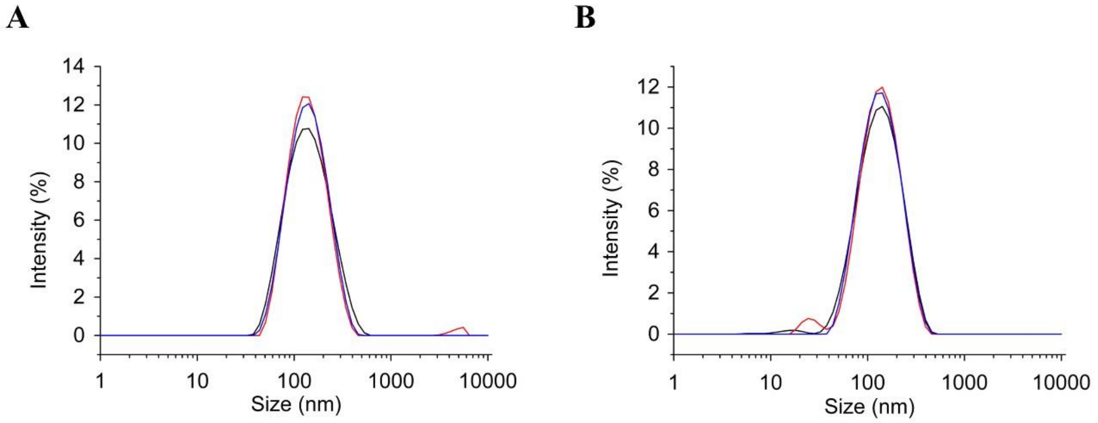

4. Isothermal titration calorimetry

Figure S3. Isothermal titration nanocalorimetry data of 1×Fluo-nanoMIP titrated with the non template protein human serum transferrin and expressed as raw heats over time.

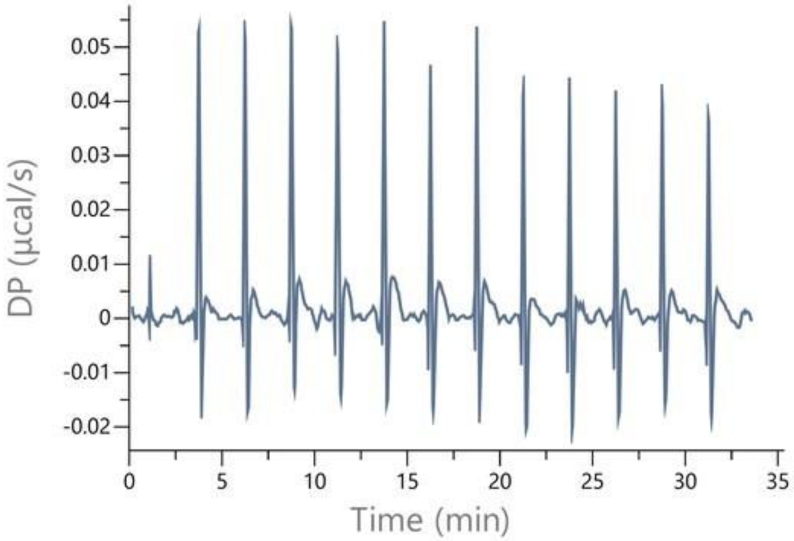

Supplement: Supplementary file 1 [file biosensors-13-00745-s001.zip › biosensors-2456871-supplementary.pdf]
